# Dr. Carnie Casander Thayer

**Published:** 1916-10

**Authors:** 


**Dr. Carnie Casander Thayer,** Rush 1878, died in the Clifton Springs Sanitarium, from gastric and duodenal ulcer, . . . . . During the Civil War, he served for two years on the Sanitary Commission and was subsequently a missionary in Turkey. He served for 21 years on the staff of the Clifton Springs Sanitarium.

